# Supplementary material for: Screen time exposure and academic performance, anxiety, and behavioral problems among school children
Source: PeerJ. 2025 May 8;13:e19409. doi: 10.7717/peerj.19409 (PMC12066102; doi:10.7717/peerj.19409)
Supplement: Supplemental Information 1 [file peerj-13-19409-s001.pdf]

Screen time exposure and academic performance, anxiety, behavioral problems among school children

	Item no	Recommendation	Location & line number
**Title and abstract**	1	(a) Study design is indicated in the abstract, methods section as an institution based cross-sectional study design.	*Page 1*<br>*Line # 1 – 2*
		(b) Methods and results section of the abstract provides a fair summary of the work that was done and the results that were discovered.	*Page 2*<br>*Methods and results: lines 44 - 55*
**Introduction**			
Background/rationale	2	The scientific background and rationale for conducting this study is reported under background section in this manuscript.	*Pages 3-4*<br>*Lines: 65-95*
Objectives	3	Specific objectives and the need for this study are stated in the last paragraph under background section.	*Page 4*<br>*Lines 96-103*
**Methods**			
Study design	4	This study used web-based cross-sectional design and the same is mentioned in 1st paragraph of method section.	*Methods section*<br>*Page 4, line 106-107.*
Setting	5	A detailed description of the study area, sample catchment area, location was described in the 2nd paragraph of method section.	*Methods section*<br>*Page 4, lines 115-116*
Participants	6	Eligibility criteria of the study participants are clearly stated in the 2nd-3rd paragraph of methods section.	*Methods section*<br>*Page 4, lines 116-117*
Variables	7	Major outcomeScreen time exposure, Spencer Children Anxiety Scale (SCAS - Child), Pediatric Symptom Checklist (PSC - Parent Version) and other predictor variables are operationally defined and mentioned under data collection tools and procedures, in the last paragraph of methods.	*Methods section*<br>*Page 5, Line 126- 141*

Data sources/ measurement	8	Source of data and data analysis methods are discussed in the data processing and analysis section.	*Data analysis section, Page 5-6 Lines 147 – 156*
Bias	9	Several sections of the methods part described efforts to address possible bias sources.	*Methods section: Page 4 -5, Lines 114-145*
Study size	10	Parents of the selected schools were conveniently recruited to participate in the web-based sirvey	*Methods section: Page 4 -5, Lines 114-119*
Quantitative variables	11	All quantitative variables are treated as qualitative after categorizing them in one of the most commonly used categories and category used are mentioned under data processing and analysis sub-section.	*Data analysis section, Page 5-6 Lines 147 – 156*
Statistical methods	12	(*a*) Statistical methods used in this study are described under data analysis sub-section in the paragraph of data processing and analysis section.	*Data analysis section, Page 5-6 Lines 147 – 158*
		(*b*) Both sub group analysis and interaction terms were used.	*Data analysis section, Line 156-157*
		(*c*) There were no missing data in this study	*Data analysis section, Page 6, line 158*
		(d) Not applicable	
		(*e*) Not applicable	
**Results**			
Participants	13	(a) details socio-demographic and behavioral characteristics in the table 1 and anxiety, behavioural outcome were presented in table 2	*Results section, Page 6, lines 161-170*
		(b) This was cross-sectional study so; there is no flow as that of longitudinal study	*NA*
**Descriptive data**	14	(a) Characteristics of study participants (eg demographic,) and information on screen time exposures and factor associated is presented in tables 1.	*Results section, Page 6, lines 161-170*
		(b) There were no missing data in this study	*No missing data*
Outcome data	15	Spencer Children Anxiety Scale (SCAS - Child)	*Results section, page 6, lines 167-170.*

		Pediatric Symptom Checklist (PSC - Parent Version)	
**Main results**	16	(*a*) Unadjusted estimates and confounder-adjusted estimates and their precision (e.g., 95% confidence interval) are presented in table 3. Discussed in 1st paragraph under regression analysis of result section.	*Results section, page 7, lines 199-202*
		(b) Category boundaries of continuous variables were categorized and reported in all tables.	*Results section, pages 6-7, lines 167-202.*
		(*c*) Linear Regression model was used and expressed in odds ratio.	
Other analyses	17	Correlation analysis	*Results section, page 6-7, lines 179-190*
**Discussion**			
Key results	18	Key results to study objectives are discussed under discussion session with references.	*Discussion section, page 7-8, lines 207-211*
Limitations	19	Limitations and possible strengths related to this study are discussed in the final paragraph of the discussion session on the way of viewing direction for researchers.	*Discussion section, page 9-10, lines 264-271.*
Interpretation	20	A cautious overall interpretation of results considering objectives, results from similar studies, and other relevant evidence is discussed under the limitation of discussion session.	*Discussion section, page 9-10, lines 213-262*
Generalizability	21	The generalizability (external validity) of the study results are mentioned in the paragraph of discussion section and in the conclusion section.	*Page 11, Lines 275-277, 310-314*
**Other information**			
Funding	22	Information regarding the source of funding Prince Sattam bin Abdulaziz University project number (PSAU/2024/R/1445), Saudi Arabia are presented in the declaration section	*In declaration section*
